# Association of Serum Selenium with Clinical Features and Inflammatory and Oxidative Stress Markers in Iranian Patients with Metabolic Dysfunction-Associated Steatotic Liver Disease—A Cross-Sectional Study

**DOI:** 10.3390/diagnostics15121559

**Published:** 2025-06-18

**Authors:** Abbas Pishdadian, Reza Sharifi, Adele Shafaghi, Soudabeh Hamedi-Shahraki, Farshad Amirkhizi, Aleksandra Klisic

**Affiliations:** 1 Department of Immunology, Faculty of Medicine, Zabol University of Medical Sciences, Zabol 98616-15881, Iran; 2 Student Research Committee, Faculty of Medicine, Zabol University of Medical Sciences, Zabol 98616-15881, Iran; 3 Department of Epidemiology and Biostatistics, Faculty of Public Health, Zabol University of Medical Sciences, Zabol 98616-15881, Iran; 4 Department of Nutrition, Faculty of Public Health, Zabol University of Medical Sciences, Zabol 98616-15881, Iran; 5 Department of Biochemistry, Faculty of Medicine, University of Montenegro, 81000 Podgorica, Montenegro; 6 Center for Laboratory Diagnostics, Primary Health Care Center, 81000 Podgorica, Montenegro

**Keywords:** metabolic dysfunction-associated steatotic liver disease, selenium, inflammation, oxidative stress, insulin resistance

## Abstract

**Background**: There are conflicting epidemiological studies regarding the association between selenium (Se) and metabolic disorders. Furthermore, the pathophysiological links between Se and metabolic dysfunction-associated steatotic liver disease (MASLD) have not yet been fully elucidated. Therefore, we evaluated the association between serum Se levels and the clinical features of MASLD and the inflammatory and oxidative stress markers in these patients as potential risk factors for the progression of this disease. **Methods**: This cross-sectional study involved 150 patients aged 20 to 50 years who were newly diagnosed with MASLD. Oxidative stress was evaluated by measuring serum thiobarbituric acid reactive substances (TBARS), total antioxidant capacity (TAC), and the activities of erythrocyte superoxide dismutase (SOD) and glutathione peroxidase (GPx). Tumor necrosis factor-alpha (TNF-α), interleukin-6 (IL-6), and transforming growth factor beta (TGF-β) were measured as inflammatory markers. A one-way analysis of variance (ANOVA), Pearson chi-square test, Kruskal–Wallis test, and multiple linear regression were employed for data analysis. **Results**: We observed a significant inverse association between serum Se concentrations and liver steatosis severity in the participants. There was a significant decrease in serum concentrations of insulin and the homeostasis model assessment of insulin resistance (HOMA-IR), triglycerides, TNF-α, and TBARS with ascending quartiles of serum Se. Conversely, the mean serum levels of TAC and erythrocyte GPx activities exhibited a consistent increasing trend in relation to rising serum Se concentrations. However, no significant trends were identified for serum FSG, IL-6, TGF-β, or erythrocyte SOD activities across the varying levels of serum Se. **Conclusions**: Our results demonstrate that decreased serum selenium levels in Iranian patients with MASLD correlate with elevated inflammatory markers, increased oxidative stress, and more severe liver steatosis.

## 1. Introduction

Metabolic dysfunction-associated steatotic liver disease (MASLD), previously called non-alcoholic fatty liver disease (NAFLD), is the most widespread chronic liver condition associated with metabolic disorders, especially obesity and type 2 diabetes, affecting more than 30% of the adult population worldwide [1]. Over the past decade, the prevalence of this disease has risen significantly, likely due to lifestyle shifts that include decreased physical activity and altered dietary patterns [2]. A meta-analysis indicated that approximately 34% of the adult population in Iran is affected by MASLD [3], which is somewhat higher than the global prevalence.

The etiological mechanisms of MASLD are influenced by various metabolic, genetic, lifestyle, and dietary factors, many of which are yet to be fully understood [4]. It is well documented that the progression of MASLD is primarily driven by the deposition of fat in the liver, a process mainly caused by insulin resistance and mitochondrial dysfunction [5,6]. Additionally, oxidative stress and inflammatory cytokines are identified as key contributors to the onset and progression of MASLD [7,8]. Therefore, deficiencies in nutrients that have antioxidant, anti-inflammatory, and insulin-sensitizing properties may play a role in the development of MASLD [7,8].

Selenium (Se) is an essential trace element that serves as a crucial component in the active sites of several proteins, such as glutathione peroxidase (GPx), thioredoxin reductase, selenoprotein P, and iodothyronine deiodinase [9,10]. This essential micronutrient plays a role in a wide range of bodily functions, including cell signaling, protection against free radicals, the regulation of inflammatory responses, and the proper functioning of the immune and reproductive systems [9,11,12]. Consequently, the many health benefits Se brings about by supporting overall human health are likely due to its ability to suppress pro-inflammatory cytokines and neutralize reactive oxygen and nitrogen species [13].

Diet is the primary source of Se intake, and the levels of this trace element in foods, especially plant-based ones, depend on the Se content of the soil in different regions; therefore, dietary Se intake varies widely across countries [14,15]. Although determining appropriate reference values is useful for the interpretation and clinical management of Se disorders, these values vary among different populations and should be established regionally [16]. Unfortunately, reference values have not yet been determined in Iran. However, serum Se concentrations ranging from 70 to 100 μg/L (0.9 to 1.3 μmol/L) have been suggested by various authors to indicate “Se adequacy,” while levels below 70 μg/L are considered indicative of “Se deficiency” in European adults [16].

Research has shown that the Se content in soil and water across many regions of Iran is significantly low [17]. In the liver, selenomethionine is broken down into selenide, which is then used to produce selenoproteins, such as selenoprotein P, and enzymes like GPx. These compounds are essential for protecting the body against oxidative stress due to their antioxidant functions [18,19].

There are conflicting epidemiological studies regarding the association between Se and metabolic disorders. While some studies suggest that a higher Se intake and elevated blood Se levels are associated with an increased risk of diabetes [20], dyslipidemia [21], and hypertension [22], the evidence remains inconsistent. However, only a limited number of epidemiologic studies have examined the relationship between Se and the risk of developing MASLD [23,24,25]. The results of these studies have been inconsistent. Furthermore, serum Se levels in patients with MASLD and cirrhosis have been shown to be lower [26,27].

However, the pathophysiological links between Se and MASLD have not yet been fully elucidated, and the existing studies remain inconclusive. Furthermore, the relationship between Se and insulin resistance, a key pathogenic factor in MASLD, is also unclear. To bridge this gap in our understanding, we carried out a cross-sectional study to investigate the relationships between serum Se levels and markers of insulin resistance, inflammation, and oxidative stress, as well as clinical features, in Iranian patients diagnosed with MASLD within the Iranian population.

## 2. Materials and Methods

### 2.1. Participants

**Figure 1:**
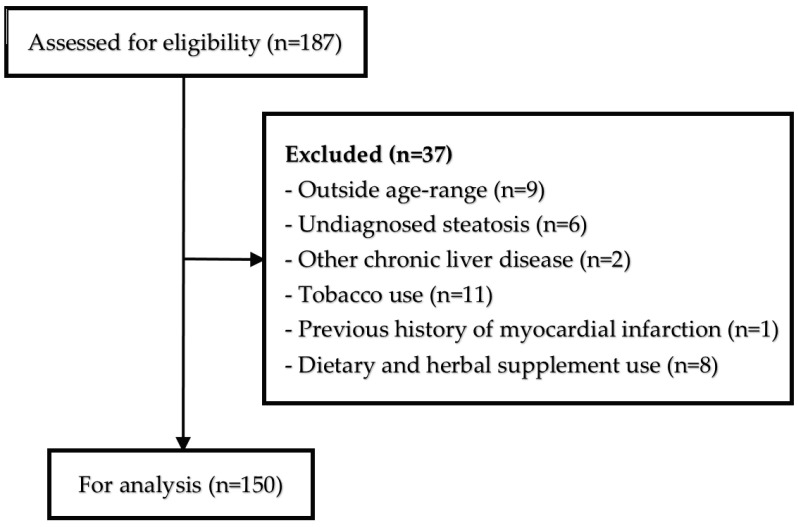
Flowchart of the participants’ selection.

This cross-sectional study included 150 patients, aged 20 to 50 years, who had been recently diagnosed with MASLD. The participants were selected from outpatient referrals to the Sheykholrayis polyclinic in Tabriz, Iran, during the period from June to December 2024. The flowchart of the study participants’ selection is shown in Figure 1.

The liver ultrasound was conducted while patients were in a fasting state by a single radiologist using a SonoAce X4 ultrasound system (Sonoace X4 Medisio, Seoul, Republic of Korea). The severity of liver steatosis was evaluated by a qualified ultrasonographer using the grading system established by Hamaguchi et al. [28] and classified into three categories: grade 1, grade 2, and grade 3.

Grade 0 indicates normal liver echotexture. However, since our cohort encompassed only patients with liver steatosis, they were divided into 3 categories, which are as follows: grade 1 indicates a diffuse and slight increase in liver echogenicity with normal visualization of the portal vein wall and the diaphragm; grade 2 indicates a moderate increase in liver echogenicity with a slightly impaired appearance of the diaphragm and the portal vein wall; and grade 3 indicates a marked increase in liver echogenicity with poor or no visualization of the diaphragm, portal vein wall, and posterior part of the right liver lobe. Participants were classified as having MASLD if they exhibited liver steatosis along with at least one of the following metabolic criteria [29]: a body mass index (BMI) of ≥25 kg/m^2^ or a waist circumference (WC) of ≥94 cm in men and ≥80 cm in women; fasting serum glucose (FSG) of ≥100 mg/dL (≥5.6 mmol/L) or glycated hemoglobin (HbA1c) of ≥5.7%, a diagnosis of type 2 diabetes mellitus, or treatment for type 2 diabetes mellitus; systolic blood pressure (SBP) of ≥130 mm Hg, diastolic blood pressure (DBP) of ≥85 mm Hg, or treatment with antihypertensive medications; serum triglycerides (TG) of ≥150 mg/dL (≥1.70 mmol/L) or treatment with lipid-lowering medications; and serum high-density lipoprotein cholesterol (HDL-C) of ≤40 mg/dL (≤1.0 mmol/L) for men or ≤50 mg/dL (≤1.30 mmol/L) for women, or treatment with lipid-lowering medications.

This study’s exclusion criteria encompassed several specific conditions: participants who were pregnant or breastfeeding; individuals diagnosed with liver disease other than MASLD; and those with notable organic or inflammatory disorders, a documented history of stroke or myocardial infarction, who were administered medications affecting inflammation within the six months preceding the study, who took any herbal or dietary supplements for a minimum of six months prior to participation, or who used tobacco and alcohol.

This study was conducted in accordance with the Declaration of Helsinki, and the study protocol received approval from the ethics committee of Zabol University of Medical Sciences (Reference number: IR.ZBMU.REC.1403.009). All participants were asked to provide written informed consent following a thorough explanation of the study’s objectives and protocol.

### 2.2. Physical Activity, Anthropometric, and Body Composition Measurements

Body weight was assessed utilizing a standard digital weighing scale (SECA-Germany, Hamburg, Germany) and documented to the nearest 0.1 kg. Height was measured in an upright posture with a fixed wall scale and recorded to the nearest 0.5 cm. Patients’ BMI was computed by dividing their weight in kilograms by the square of their height in meters. A stretch-resistant tape measure was used to measure WC.

The short version of the International Physical Activity Questionnaire was utilized to assess the levels of physical activity among participants [30]. The individuals were classified into three distinct categories based on their physical activity levels: low, moderate, and vigorous.

### 2.3. Biochemical Measurements

After 12 h of overnight fasting, a 10 mL venous blood sample was collected from each patient and subsequently subjected to centrifugation at 3200 rpm for a duration of 10 min to isolate the serum. The serum activities of alanine aminotransferase (ALT), aspartate aminotransferase (AST), and gamma-glutamyl transferase (GGT); serum levels of lipid parameters [TG, HDL-C, low-density lipoprotein cholesterol (LDL-C), and total cholesterol (TC)]; and FSG were assessed by an automatic analyzer (Abbott, model Alcyon 300, Chicago, IL, USA).

Serum insulin concentrations were quantified utilizing an enzyme-linked immunosorbent assay (ELISA) kit (DiaMetra, Milano, Italy). Insulin resistance was assessed through the homeostasis model assessment method (HOMA-IR), employing the following formula: HOMA-IR = [fasting insulin (U/L) × FSG (mg/dL)]/405 [31].

Serum concentrations of TNF-α, IL-6, and TGF-β were measured using appropriate ELISA kits (Crystal Day Bio-Tec, Shanghai, China). Serum thiobarbituric acid reactive substance (TBARS) concentrations were measured using a method that was initially described by Uchiyama and Mihara [32]. The serum total antioxidant capacity (TAC) of samples was assessed colorimetrically and in triplicate utilizing 2, 2′-Azino-di-[3-ethylbenzthiazoline sulphonate] (ABTS) [33]. The evaluation was predicated on the ability of serum antioxidants to inhibit the oxidation of ABTS to ABTS•+ by a peroxidase enzyme, resulting in the formation of a colored complex that exhibits peak absorption at 600 nm.

The enzymatic activity of superoxide dismutase (SOD) and glutathione peroxidase (GPx) in erythrocyte hemolysates was assessed utilizing a Ransod kit (Randox Laboratories, Ltd., Crumlin, UK, catalog number SD-125) and a Ransel kit (Randox Laboratories, Ltd., Crumlin, UK, catalog number RS-504), respectively.

Serum Se concentrations were quantified utilizing a hydride generation atomic absorption spectrophotometer (Shimadzu AA-680, Kyoto, Japan) equipped with a pyrolytically coated graphite furnace (GFA-EX7i, Shimadzu, Kyoto, Japan). The analysis was conducted using a selenium hollow cathode lamp operating at a wavelength of 196.0 nm, with a current of 23 mA, a bandpass of 0.7 nm, and a deuterium background corrector to enhance measurement accuracy.

### 2.4. Statistical Analysis

The data were analyzed using IBM SPSS version 25 (IBM Corp, Armonk, New York, NY, USA). Participants were classified based on the quartile cut-points of serum Se levels, which were as follows: Q1, <46.97; Q2, 46.97 to 57.46; Q3, 57.47 to 63.44; and Q4, >63.45. The assessment of the normality of the data’s distribution was conducted utilizing a Q-Q plot in conjunction with the Kolmogorov–Smirnov test. Quantitative data that conformed to a normal distribution were expressed as means ± standard deviation, whereas qualitative data were represented in terms of frequency (percentages). In variables where the data did not follow a normal distribution, the median and interquartile range (IQR) were employed for reporting.

In order to examine the general characteristics across the quartiles of serum Se levels, a one-way analysis of variance (ANOVA) was utilized for quantitative variables, while the Pearson chi-square test was employed for qualitative variables. For markers that did not follow a normal distribution, the non-parametric Kruskal–Wallis test was applied to assess differences among the serum Se quartiles. Additionally, in the non-normally distributed data, the independent-samples Jonckheere–Terpstra test was utilized to ascertain the *p*-trend.

To investigate the relationship between serum Se concentrations and biochemical markers, multiple linear regression analyses were conducted using both unadjusted and adjusted models. The adjusted model incorporated covariates including age, sex, BMI, and levels of physical activity. A *p*-value threshold of less than 0.05 was established to determine statistical significance.

## 3. Results

The study sample comprised 150 patients with MASLD, with a mean age of 39.2 ± 6.6 years and a mean BMI of 31.3 ± 3.5 kg/m^2^. The demographic characteristics of the participants are presented in Table 1. Their mean age, weight, BMI, and WC were consistent across the quartiles of serum Se levels. Furthermore, the distributions of education level and physical activity were comparable across the quartiles of serum Se levels.

**Figure 2:**
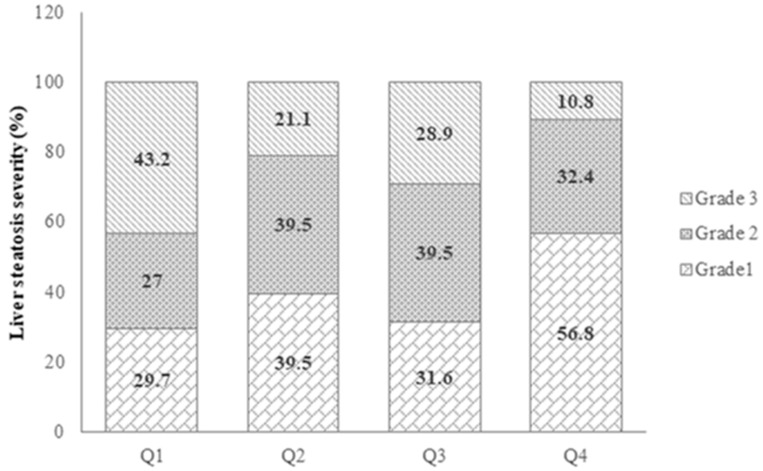
Distribution of liver steatosis severity of patients with MASLD across quartiles (Qs) of serum selenium. Number of patients in Q1, *n* = 37; Q2, *n* = 38; Q3, *n* = 38; and Q4, *n* = 37. *p*-values obtained from Mantel–Haenszel chi-square test. *p* < 0.05 was considered significant.

Figure 2 illustrates the distribution of liver steatosis severity among patients with MASLD across the quartiles of serum Se. We observed a significant inverse trend between serum Se concentrations and liver steatosis severity in the participants (*p*-trend = 0.006).

Table 2 illustrates the average serum concentrations of metabolic, inflammatory, and oxidative stress markers in patients diagnosed with MASLD across the quartiles of serum Se. The data revealed a statistically significant decreasing trend in the mean serum levels of insulin (*p*-trend = 0.005), HOMA-IR (*p*-trend = 0.002), and TG (*p*-trend = 0.014) as the serum Se quartile increased. Additionally, there was a notable decline in serum concentrations of TNF-α (*p*-trend < 0.001) and TBARS (*p*-trend = 0.025) with ascending quartiles of serum Se. Conversely, the mean serum levels of TAC (*p*-trend = 0.009) and erythrocyte GPx activities (*p*-trend = 0.017) exhibited a consistent upward trend in relation to increasing serum Se concentrations. However, no significant trends were identified for serum FSG, IL-6, TGF-β, or erythrocyte SOD activities across the varying levels of serum Se (Table 2).

**Figure 3:**
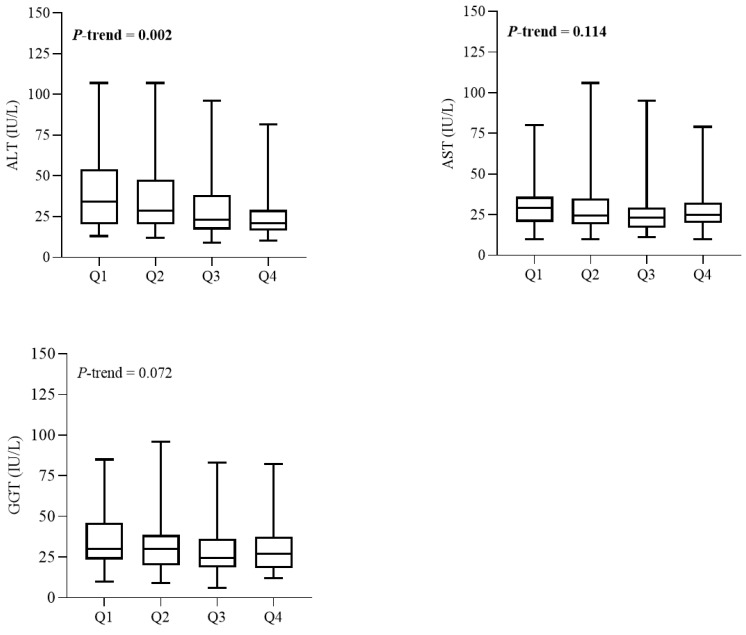
Liver enzymes levels of patients with MASLD across quartiles (Qs) of serum selenium. Number of patients in Q1, *n* = 37; Q2, *n* = 38; Q3, *n* = 38; and Q4, *n* = 37. Data are expressed as medians (with interquartile ranges). *p*-values obtained from Kruskal–Wallis test. *p* < 0.05 was considered significant. ALT, alanine aminotransferase; AST, aspartate aminotransferase; GGT, gamma glutamyl transferase.

Upon analyzing the relationships between serum Se concentrations and serum liver enzyme activities, as depicted in Figure 3, we identified a notable trend indicating a reduction in serum ALT activity with increasing quartile categories of Se (*p* = 0.006). Conversely, no significant correlations were found between serum Se levels and the serum activities of AST and GGT.

The results of the multiple linear regression analysis, which included biochemical markers as dependent variables and serum Se concentrations as the independent variable, are detailed in Table 3. In the crude model, serum Se concentrations exhibited an inverse correlation with serum levels of insulin, TG, ALT, TNF-α, TBARS, and HOMA-IR. In contrast, serum Se concentrations demonstrated a positive correlation with serum TAC levels and erythrocyte SOD activities. These relationships remained statistically significant after controlling for potential confounding variables, including age, sex, BMI, and physical activity level, in the adjusted model.

## 4. Discussion

The primary objective of this study was to investigate the association between serum Se levels and inflammatory and oxidative/antioxidative biomarkers, as well as clinical features, in patients with MASLD. The results indicated that serum concentrations of insulin, HOMA-IR, and TG decreased as serum Se levels increased. Additionally, there was a significant reduction in serum concentrations of TNF-α, TBARS, and ALT with rising serum Se concentrations. Conversely, the mean serum levels of TAC and erythrocyte GPx activities exhibited an increase with rising serum Se concentrations. However, no significant trends were identified for serum FSG, IL-6, TGF-β, AST, and GGT and erythrocyte SOD activities across the varying levels of serum Se. We also observed a significant inverse relationship between serum Se concentrations and the severity of liver steatosis in the participants. Therefore, the present findings demonstrate an association between serum Se concentrations and certain inflammatory and oxidative/antioxidative parameters, as well as the severity of liver steatosis, in patients with MASLD.

Insulin resistance is a key characteristic of MASLD and is characterized by diminished insulin sensitivity in the whole body, including the liver, skeletal muscle, and adipose tissues [34]. Evidence from studies utilizing gold-standard measures of insulin action has consistently demonstrated that liver steatosis, independent of adiposity, is associated with impaired insulin function in these tissues [35]. Nevertheless, the mechanisms underlying decreased insulin sensitivity and the subsequent accumulation of fat in the liver have not yet been fully elucidated. We hypothesized that dietary-derived agents, such as Se, may be associated with insulin resistance in patients with MASLD. We revealed an inverse association between serum Se levels and insulin resistance, as measured by the HOMA-IR levels. In accordance with our results, Kamali et al. found that an intervention of 200 µg/day of Se for four weeks significantly improved glucose metabolism by decreasing FSG, serum insulin, and HOMA-IR in patients undergoing coronary artery bypass grafting (CABG) surgery [36]. Furthermore, a 12-week Se supplementation for patients with congestive heart failure significantly reduced insulin concentrations and HOMA-IR [37]. However, in patients with metabolic syndrome, Se supplementation was not effective in reducing HOMA-IR [38]. On the contrary, a clinical trial involving individuals aged 40–80 years found no significant change in HOMA-IR after 2.9 years of Se supplementation at a dose of 200 µg/day [39]. Interestingly, a longitudinal study reported a similar finding, indicating that male participants with higher plasma Se concentrations demonstrated a reduced early insulin response during baseline intravenous glucose tolerance tests after 20 years of follow-up [40]. Several proposed mechanisms by which Se ameliorates insulin resistance include the activation of kinases involved in the insulin signaling cascade [41] and the enhancement of peroxisome proliferator-activated receptor gamma (PPAR-γ) mRNA expression [42]. PPAR-γ is a key mediator of insulin sensitivity and plays a crucial role in influencing glucose and lipid uptake in peripheral tissues [42]. Nonetheless, numerous studies have demonstrated a correlation between elevated serum Se concentrations and dietary Se intake and an increased risk of metabolic disorders, particularly diabetes [20], MASLD [23,24], and all-cause mortality. These findings support the hypothesis that selenium’s metabolic effects exhibit a U-shaped dose–response relationship [43], indicating that optimal intake is essential for health, while both a deficiency and excess can be detrimental. The favorable association between Se levels and insulin resistance in our findings could be explained by the fact that none of the samples exhibited excessive, potentially toxic, selenium concentrations. These controversial findings may be partially due to differences in the study subjects’ characteristics, the methodology of the studies, and/or sample size. Our study population may have had a lower baseline Se intake compared to studies that link selenium to harm, such as those containing U.S. and European cohorts with Se-rich diets. Furthermore, the “toxic” threshold may vary among different populations, such as those with genetic polymorphisms in selenoprotein pathways. In addition, polymorphisms in selenoproteins may affect individual responses to Se.

In recent years, a growing body of evidence has indicated that oxidative stress may play a significant role in the pathophysiology of MASLD and its progression to fibrosis and chronic liver disease [7,44]. However, one of the key unresolved questions in MASLD research is identifying the specific factors linked to increased oxidative stress in these patients. Oxidative stress is defined as a disturbance in the balance between the production of reactive oxygen species and their elimination by protective antioxidant defenses, such as enzymatic and non-enzymatic defense systems [45]. This study revealed favorable associations between serum Se concentrations and markers of oxidant/antioxidant status, including serum TBARS and TAC. Consistent with our findings, a Se supplementation in patients undergoing CABG surgery for four weeks significantly reduced plasma malondialdehyde (MDA) levels, which is an important marker of lipid peroxidation [36]. Similarly, Se supplementation in patients on hemodialysis for 3 months significantly reduced serum MDA concentrations [46]. Se is essential for the synthesis of selenium-dependent glutathione peroxidase (Se-GPx), which is crucial for combating free radical oxidation [47]. In our study, erythrocyte GPx activity was higher in patients with elevated serum Se levels compared to those with lower levels. Amirkhizi and colleagues also reported a direct association between serum Se levels and erythrocyte GPx activity in patients with polycystic ovary syndrome (PCOS) [48]. However, these researchers found no significant association between serum Se levels and erythrocyte SOD activity, which is consistent with our results. Likewise, Luo et al. observed no correlation between serum Se levels and SOD activity in patients with ischemic cardiomyopathy [49]. This result can likely be attributed to the fact that Se is not directly responsible for SOD activity. Therefore, the lower serum levels of TBARS and the higher levels of TAC observed in patients with elevated serum Se levels may be partially attributed to the increased erythrocyte GPx activity in individuals with higher serum Se concentrations.

A growing body of evidence supports the crucial role of TNF-α and other inflammatory cytokines in the progression of pure steatosis to metabolic dysfunction-associated steatohepatitis (MASH) [50]. We found an inverse association between serum Se levels and serum levels of TNF-α, whereas no such relationship was observed for serum IL-6 and TGF-β concentrations. Gholizadeh et al., in a meta-analysis of 24 randomized controlled trials, indicated that Se supplementation decreases serum IL-6 and increases serum TNF-α levels [51]. The different results of these studies might be, in part, due to the difference in the sample size of the studies, geography, and the characteristics of the populations who participated in the studies. In fact, our study population may have had different baseline Se levels compared to the pooled studies included in the meta-analysis and the aforementioned studies. The effects of Se are U-shaped, meaning they can be harmful at both deficiency and excess levels, and outcomes may vary based on the initial status of Se in the participants. In addition, participants in the studies may have exhibited varying degrees of metabolic dysfunction (e.g., diabetes, obesity) or MASLD severities, which could influence their cytokine responses.

Extensive research has established and acknowledged the significant role of oxidative stress in mediating pro-inflammatory effects [52]. Antioxidants possess the capability to mitigate inflammation and oxidative stress at the level of gene expression [53]. It has been demonstrated that Se inhibits the activation of nuclear factor kappa-B (NF-κB) and modulates cytokine production at the gene expression level by reversibly disrupting the formation of disulfide bridges, resulting in a decrease in inflammatory markers, including TNF-α [54].

Our study has several strengths and potential limitations that need to be considered. The panel of biomarkers utilized in this study was fairly comprehensive in assessing both oxidative stress and inflammation. Furthermore, several potential confounders, including age, sex, BMI, and physical activity level, were adjusted in our analyses. However, the current study had some limitations that should be considered. These results could be influenced by unrecognized confounders, as is the case with all observational studies. We also were unable to assess the dietary intake of the participants, particularly their antioxidant nutrient intake. Additionally, causality could not be assessed due to the cross-sectional design of this study. The lack of a control group and a relatively small sample size point to the need for future studies with a longitudinal design and a larger sample size that include a non-MASLD control group to overcome these limitations.

## 5. Conclusions

In conclusion, our study supports the notion that low serum levels of Se in Iranian patients with MASLD are associated with elevated levels of serum TBARS and reduced levels of serum TAC, as well as decreased erythrocyte GPx activity. In addition, an inverse association was found between the serum levels of Se and serum levels of TNF-α, which serves as an inflammatory marker. Indeed, these findings confirm the anti-inflammatory and antioxidant properties of Se and its potential roles in the prevention of MASLD progression and the improvement of clinical features. However, further long-term prospective studies involving larger cohorts of patients with biopsy-proven MASLD are necessary to confirm these findings and to ascertain whether improvements in serum Se levels in those with MASLD will ultimately prevent or delay the development and progression of the disease.

## Figures and Tables

**Table 1:** General characteristics of patients with MASLD across quartiles of serum selenium levels ^a^.

Variables		Total (*n* = 150)	Quartiles of Serum Selenium				*p* ^b^
			Q1 (*n* = 37)	Q2 (*n* = 38)	Q3 (*n* = 38)	Q4 (*n* = 37)	
Male, *n* (%)		77 (51.3)	14 (37.8)	18 (47.4)	25 (65.8)	20 (54.1)	0.101
Age (years)		39.2 ± 6.6	38.3 ± 6.2	40.1 ± 6.5	40.0 ± 6.3	38.1 ± 7.3	0.393
Weight (kg)		87.0 ± 11.9	85.1 ± 9.7	85.7 ± 12.1	91.2 ± 13.8	86.0 ± 11.2	0.093
BMI (kg/m^2^)		31.3 ± 3.5	31.3 ± 3.4	30.9 ± 3.3	31.4 ± 3.0	31.5 ± 4.2	0.864
Waist circumference (cm)		101.8 ± 9.1	99.9 ± 8.5	100.3 ± 8.7	103.8 ± 7.8	103.2 ± 10.7	0.137
Education level, *n* (%)							
	<Diploma	85 (56.7)	25 (67.6)	22 (57.9)	21 (55.3)	17 (45.9)	0.311
	Diploma and university	65 (43.3)	12 (32.4)	16 (42.1)	17 (44.7)	20 (54.1)	
Physical activity level, *n* (%)							
	Low	107 (71.3)	28 (75.7)	26 (68.4)	25 (65.8)	28 (75.7)	
	Moderate	32 (21.3)	8 (21.6)	9 (23.7)	8 (21.1)	7 (18.9)	0.724
	Vigorous	11 (7.3)	1 (2.7)	3 (7.9)	5 (13.2)	2 (5.4)	
Serum Se (µg/L)		55.4 ± 12.1	38.5 ± 6.7	52.9 ± 2.8	60.6 ± 1.8	69.4 ± 4.5	<0.001

MASLD, metabolic dysfunction-associated fatty liver disease; Se, selenium. ^a^ Data are shown as means ± standard deviation for continuous variables and numbers (%) for categorical variables. ^b^ Obtained from one-way ANOVA for continuous variables and chi-square test for categorical variables.

**Table 2:** Metabolic, inflammatory, and oxidative stress markers in patients with MASLD across quartiles of serum selenium.

Variables	Quartiles of Serum Selenium				*p*-Trend
	Q1 (*n* = 37)	Q2 (*n* = 38)	Q3 (*n* = 38)	Q4 (*n* = 37)	
FSG (mg/dL)	106.0 ± 13.6	102.7 ± 10.3	104.5 ± 16.1	99.9 ± 10.0	0.078 ^a^
Insulin (µU/mL)	18.0 ± 9.6	14.5 ± 8.5	15.2 ± 9.2	11.5 ± 8.2	0.005 ^a^
HOMA-IR	4.9 ± 3.2	3.7 ± 2.2	4.0 ± 2.5	2.9 ± 2.0	0.002 ^a^
TC (mg/dL)	199.3 ± 31.5	199.7 ± 25.3	195.5 ± 41.2	209.7 ± 33.5	0.272 ^a^
LDL-C (mg/dL)	127.0 ± 19.4	125.8 ± 25.8	127.5 ± 32.2	124.4 ± 39.7	0.790 ^a^
HDL-C (mg/dL)	47.0 ± 8.5	46.0 ± 10.8	42.0 ± 10.3	44.2 ± 13.1	0.120 ^a^
TG (mg/dL)	213.8 ± 76.1	201.2 ± 75.5	161.7 ± 57.1	185.6 ± 60.3	0.014 ^a^
TNF-α (pg/mL)	28.3 (24.4, 37.6)	26.8 (22.7, 29.6)	27.0 (22.9, 28.9)	24.1 (19.8, 27.5)	<0.001 ^b^
IL-6 (pg/mL)	5.70 (4.56, 7.55)	5.20 (4.37, 6.85)	4.87 (4.36, 5.70)	4.95 (4.40, 6.26)	0.113 ^b^
TGF-β (ng/mL)	641.5 (364.5, 744.6)	702.9 (422.0, 878.8)	651.0 (476.8, 793.0)	688.4 (503.6, 812.4)	0.318 ^b^
TBARS (nmol/mL)	1.97 (1.68, 2.41)	1.90 (1.69, 2.24)	1.85 (1.63, 2.04)	1.82 (1.58, 2.03)	0.025 ^b^
TAC (mmol/L)	1.42 (1.23, 1.62)	1.53 (1.26, 1.78)	1.58 (1.28, 2.08)	1.65 (1.37, 2.24)	0.009 ^b^
SOD (U/gHb)	1139 (1070, 1225)	1172 (1080, 1297)	1160 (1060, 1286)	1171 (1077, 1298)	0.566 ^b^
GPx (U/gHb)	38.2 (30.5, 47.0)	41.3 (32.7, 51.1)	43.0 (34.0, 51.1)	44.8 (36.0, 54.4)	0.017 ^b^

MASLD, metabolic dysfunction-associated fatty liver disease; FSG, fasting serum glucose; HOMA-IR, homeostasis model assessment of insulin resistance; TC, total cholesterol; LDL-C, low-density lipoprotein cholesterol; HDL-C, high-density lipoprotein cholesterol; TG, triglycerides; TNF-α, tumor necrosis factor-α; IL-6, interleukin-6; TGF-β, transforming growth factor beta; TBARS, thiobarbituric acid reactive substances; TAC, total antioxidant capacity; SOD, superoxide dismutase; GPx, glutathione peroxidase. Data are presented as mean ± standard deviation for normally distributed variables and as IQR for non-normally distributed variables. ^a^ Obtained from one-way ANOVA test. ^b^ Obtained from Kruskal–Wallis test.

**Table 3:** Results of a multiple linear regression analysis that evaluated the association between serum selenium and biochemical markers in patients with MASLD (*n* = 150).

Variables	Crude Model			Adjusted Model ^a^		
	*β*	S.E._β_	*p*-Value ^b^	*β*	S.E._β_	*p* ^b^
FSG (mg/dL)	−0.11	0.08	0.149	−0.12	0.08	0.142
Insulin (µU/mL)	−0.29	0.11	0.008	−0.30	0.11	0.006
HOMA-IR	−1.05	0.37	0.005	−1.10	0.37	0.004
TC (mg/dL)	0.01	0.03	0.657	0.02	0.03	0.581
LDL-C (mg/dL)	−0.02	0.03	0.555	−0.02	0.03	0.572
HDL-C (mg/dL)	−0.15	0.09	0.110	−0.12	0.09	0.198
TG (mg/dL)	−0.04	0.01	0.012	−0.03	0.01	0.020
ALT (IU/L)	−0.14	0.04	0.002	−0.15	0.04	0.001
AST (IU/L)	−0.08	0.07	0.249	−0.09	0.07	0.205
GGT (IU/L)	−0.08	0.05	0.174	−0.09	0.06	0.122
TNF-α (pg/mL)	−0.18	0.08	0.023	−0.16	0.08	0.037
IL-6 (pg/mL)	−0.40	0.26	0.136	−0.38	0.26	0.155
TGF-β (ng/mL)	−0.002	0.002	0.122	0.003	0.002	0.102
TBARS (nmol/mL)	−4.74	1.89	0.014	−4.59	1.90	0.017
TAC (mmol/L)	5.41	1.77	0.003	5.48	1.77	0.002
SOD (U/gHb)	0.004	0.006	0.539	0.004	0.006	0.477
GPx (U/gHb)	0.23	0.08	0.003	0.22	0.08	0.004

MASLD, metabolic dysfunction-associated fatty liver disease; FSG, fasting serum glucose; HOMA-IR, homeostasis model assessment of insulin resistance; TC, total cholesterol; LDL-C, low-density lipoprotein cholesterol; HDL-C, high-density lipoprotein cholesterol; TG, triglycerides; ALT, alanine aminotransferase; AST, aspartate aminotransferase; GGT, gamma glutamyl transferase; TNF-α, tumor necrosis factor-α; IL-6, interleukin-6; TGF-β, transforming growth factor beta; TBARS, thiobarbituric acid reactive substances; TAC, total antioxidant capacity; SOD, superoxide dismutase; GPx, glutathione peroxidase; S.E.β, standard error of β (regression coefficients). ^a^ Adjusted for age, sex, BMI, and physical activity level. ^b^ *p* < 0.05 was considered significant.

## Data Availability

The datasets used and/or analyzed during the current study are available from the corresponding authors on reasonable request.

## Abbreviations

MASLD: metabolic dysfunction-associated steatotic liver disease; Se: selenium; TBARS: thiobarbituric acid reactive substances; TAC: total antioxidant capacity; SOD: superoxide dismutase; GPx: glutathione peroxidase; TNF-α: tumor necrosis factor-alpha; IL-6: interleukin-6; TGF-β: transforming growth factor beta; FSG: fasting serum glucose; HOMA-IR: homeostasis model assessment of insulin resistance.
